# Screening of heat stress-regulating active fractions in mung beans

**DOI:** 10.3389/fnut.2022.1102752

**Published:** 2023-02-20

**Authors:** Yuchao Feng, Xia Fan, Dengcheng Suo, Shu Zhang, Yantao Ma, Haoyu Wang, Xin Guan, Hongzhi Yang, Changyuan Wang

**Affiliations:** ^1^College of Food, Heilongjiang Bayi Agricultural University, Daqing, China; ^2^Institute of Quality Standard and Testing Technology for Agro-Products, Chinese Academy of Agricultural Sciences, Beijing, China; ^3^Chinese National Engineering Research Center, Daqing, China

**Keywords:** heat stress, polyphenols, cells, HSP70, mung bean, UHPLC-QE-HF-HRMS

## Abstract

**Introduction:**

Heat stress caused by high temperatures has important adverse effects on the safety and health status of humans and animals, and dietary interventions to alleviate heat stress in daily life are highly feasible.

**Methods:**

In this study, the components of mung bean that have heat stress-regulating effects were characterized by in vitro antioxidant indicators and heat stress cell models.

**Results:**

As a result, 15 target monomeric polyphenol fractions were identified based on untargeted analysis on an ultra performance liquid chromatography coupled with high field quadrupole orbit high resolution mass spectrometry (UHPLC-QE-HF-HRMS) platform and available reports. The results of DPPH and ABTS radical scavenging showed that mung bean polyphenols (crude extract) and 15 monomeric polyphenols had better antioxidant activity, followed by oil and mung bean peptides, while protein and polysaccharides had relatively poor antioxidant activity. Qualitative and quantitative assays for 20 polyphenols (15 polyphenols and 5 isomers) were then established based on platform targets. Vitexin, orientin, and caffeic acid were identified as monomeric polyphenols for heat stress control in mung beans based on their content. Finally, mild (39°C), moderate (41°C), and severe (43°C) heat stress models were successfully constructed based on mouse intestinal epithelial Mode-k cells and human colorectal adenocarcinoma Caco-2 cell lines, all with an optimal heat stress modeling time of 6 h. Screening of mung bean fractions using HSP70 mRNA content, a key indicator of heat stress. As a result, HSP70 mRNA content was significantly up-regulated by different levels of heat stress in both cell models. The addition of mung bean polyphenols (crude extract), vitexin, orientin, and caffeic acid resulted in significant down-regulation of HSP70 mRNA content, and the higher the level of heat stress, the more significant the regulation effect, with orientin having the best effect. Mung bean proteins, peptides, polysaccharides, oils and mung bean soup resulted in increased or no change in HSP70 mRNA levels after most heat stresses.

**Discussion:**

The polyphenols were shown to be the main heat stress regulating components in mung bean. The results of the validation experiments confirm that the above three monomeric polyphenols may be the main heat stress regulating substances in mung bean. The role of polyphenols in the regulation of heat stress is closely linked to their antioxidant properties.

## Highlights

- Studies have confirmed that polyphenols are the main heat stress regulating component in mung beans, and that both flavonoids and phenolic acids have heat stress regulating effects.
- Orientin, vitexin and caffeic acid may be the main heat stress-regulating components in mung bean multi-fraction, with orientin being the most effective.
- The study was based on Mode-k cells and Caco-2 cells to model mild, moderate, and severe heat stress, respectively, with a modelling time of 6 h.
- A qualitative and quantitative method for the determination of 20 polyphenols, based on the UHPLC-QE-HF-HRMS platform, was targeted and found to be high in vitexin/isovitexin orientin and caffeic acid in mung beans.

## 1. Introduction

Heat is an important environmental stressor. Heat stress is the sum of non-specific responses that occur in humans or animals to excessive temperature stimuli that exceed their thermoregulatory capacity (1). Previous research has shown that the effects of heat stress is expected to be more severe in the future and that adaptation to increasing heat stress in urban areas is highly necessary (2, 3). Excessive heat exposure during daily activities can have harmful effects on the health of the body, such as heat stroke and heat cramps in humans, which can lead to death in severe cases (4). Decreased animal performance, death, and even outbreaks of various infectious diseases (5, 6). This shows that heat stress also has an important impact on the safety and health of the livestock and within poultry economic industries, food, and the environment (7). Therefore, reducing the impact of heat stress on humans and animals has become a hot topic in scientific research.

Safer and more effective dietary interventions to reduce heat stress in the food sector are highly feasible. The prevention of heat stress in animals is mostly done by adding polyphenols, vitamins, trace elements, etc. to the basic feed. Most of the heat stress studies related to humans comprise investigative reports and a few *in vivo* tests, however, no studies on dietary interventions have been reported. The intensity and duration of high temperatures have increased significantly in recent years, making it necessary to take precautions against heat stress in daily life. Mung beans are a traditional and recognized food for the prevention and elimination of heat stroke and are a preferred ingredient for dietary interventions to relieve heat stress. Mung beans also contain high levels of polyphenols, although some studies have shown that flavonoids in mung beans have heat stress-modulating effects (8). However, similar studies are rare and need to be supported by a large amount of data. Additionally, the scientific basis for the reduction of heat stress by mung beans and the primary components responsible for their effectiveness are still unclear. Heat stress as an inducer of oxidative stress has also been shown (9–12). Polyphenols have received renewed attention in recent years for their good anti-stress effects in animal models and human trials (13, 14). However, there are few reports of interventional stress studies using polyphenols in humans or epidemiological studies on the relationship between dietary polyphenol intake and stress. Cellular peroxidation has also been a focus of research in recent years in the pharmacological management of heat stroke, both nationally and internationally. It is therefore conjectured that the heat stress-regulating effect of mung beans is closely related to their high polyphenol content.

The digestion and absorption of the intestinal tract is necessary to prevent and control heat stress through dietary intervention. The intestinal tissues are also sensitive to heat stress (15), especially when animals are exposed to high temperatures for long periods of time without alleviation. Therefore, it is of practical significance to investigate the components of heat stress regulation at the cell level within the intestinal tract. Under high temperature environment, the integrity of the intestinal mucosa is disrupted, resulting in oxidative stress and massive apoptosis of the intestinal cells. Heat shock proteins (HSPs) are highly conserved proteins that are rapidly synthesized to protect the body under heat stress conditions and play a key role in the survival of stressed cells and in the repair and protection of the internal environment (16). Heat stress induces the accumulation of HSP70 in cells and is a marker of thermal tolerance in animals and cellular models (17). It can be used as a key indicator of heat stress regulation effect.

*In summary*, this study will screen the heat stress regulatory components in mung bean through *in vitro* antioxidant index and intestinal heat stress cell model to identify their main components and provide a theoretical basis for the mitigation and prevention of heat stress in mung bean.

## 2. Materials and methods

### 2.1. Materials

Plant material: mung beans comprised Ming mung beans from Shanxi, a geographical indication product of China, which were samples were collected in 2021, full-grained, and free of pests and diseases. Mung bean powder was sieved through an 80 mesh and defatted with petroleum ether for the next step of the experiment.

The mouse intestinal epithelial MODE-K cells and human colorectal adenocarcinoma Caco-2 cell line used in this experiment were provided by the College of Animal Science and Technology, Heilongjiang Bayi Agricultural University, China.

### 2.2. Experimental apparatus and reagents

SW-CJ-1FDG ultra-clean bench, LAYTE; BPN-150CH(UV) CO_2_ incubator, bluepard; MF52-N inverted microscope, Mshot; L3-5K low-speed centrifuge, Kecheng; HH-2 water bath, Changzhou Aohua Instruments Co. FlexStation 3 Multifunctional Enzyme Labeler, Molecular Devices; H1650-W Centrifuge, Xiang Yi; Tissulyser-24 Tissue Grinder, Shanghai Jingxin; 752 Spectrophotometer, Shanghai Q EXACTIVE HF, Heraeus Fresco Centrifuge, Thermo Fisher Scientific; Smart Sample Grinder, Beijing Newport Biotechnology Co., Ltd.; PS-60AL Ultrasonic Cleaner, Shenzhen Redbone Electronics Co. A TU-1800 UV-Visible Spectrophotometer from Beijing Pu-Analysis Instrument Co.

DMEM, penicillin-streptomycin solution (double antibody, 100 ×), and 0.25% trypsin solution (containing EDTA, dissolved in PBS) were purchased from Procell; Fetal Bovine Serum, ExCell Bio; MTT, MCE; cell culture dishes, cell culture bottles, Corning; malondialdehyde (MDA) test kit, lactate Total Antioxidant Capacity (T-AOC), and Superoxide Dismutase (SOD) kits were purchased from Nanjing Jiancheng Institute of Biological Engineering; Mitochondrial Membrane Potential Assay Kit, Biyuntian; methanol, acetonitrile, formic acid, purified water; all reagents were LC-MS grade and all other chemical reagents were analytically pure.

### 2.3. Mung bean fraction extraction methods

#### 2.3.1. Mung bean protein, peptide, polysaccharide, and oil extraction methods

Mung bean total protein (18), mung bean globulin (19), mung bean trypsin-hydrolyzed peptide, mung bean pepsin-pepsin-hydrolyzed peptide (20), mung bean polysaccharide (21), mung bean oil (22), and mung bean soup were prepared in the laboratory by referring to the relevant literature and making some improvements. The detailed extraction procedure is shown in Supplementary Appendix—Experimental methods.

#### 2.3.2. Mung bean polyphenol extraction method

Three grams of defatted mung bean flour was weighed and extracted with 80% ethanol at a ratio of 1:10 for 60 min under ultrasonic conditions at 40°C and 400 W. The supernatant was collected after centrifugation at 10,000 r/min for 5 min, and the residue was extracted twice as described above, for a total of three times. The ethanol was removed from the supernatant at 45°C by rotary evaporation under reduced pressure to obtain a polyphenol extract. The residue of the free phenol extraction was collected and hydrolyzed in 2 mol/L NaOH solution for 1 h. The pH was adjusted to between 2 and 3 with hydrochloric acid and then extracted three times with ethyl acetate. The supernatant was centrifuged, evaporated to until dry, and dissolved in methanol to obtain the extract of bound phenols. The free phenol and bound phenol extracts were combined and fixed to 10 ml, freeze-dried, and stored in a refrigerator at −80°C (23).

### 2.4. Antioxidant test

#### 2.4.1. DPPH free radical scavenging rate measurement

A total of 3.9 mL of DPPH was added to a working solution to 0.1 mL of the solution to be measured (the absorbance of DPPH working solution was 0.70 ± 0.02 at this time), this was shaken well and left to stand for 30 min away from light. Anhydrous ethanol was chosen as the blank reference, and absorbance value A1 was measured at 515 nm. Anhydrous ethanol (0.1 mL) was added to 3.9 mL of the DPPH working solution, and the absorbance value A2 was measured under the same conditions: 0.1 mL of the solution to be measured was added to 3.9 mL of anhydrous ethanol, and the absorbance value marked as A3 was measured under the same conditions. The DPPH radical-scavenging rate was calculated as follows:


$$DPPH\left(\%\right)=\left(1-\frac{A\,1-A\,3}{A\,2}\right)\times 100\%$$

#### 2.4.2. ABTS free radical scavenging assay

The method of Lahouar et al. (24) was used for determination, with slight modifications. The prepared ABTS^+^ stock solution was incubated overnight protected from light and diluted with ethanol before use to give an absorbance value of 0.70 ± 0.02 at 734 nm. Zero point one milliliters of the sample solution was mixed with 3.9 mL of freshly prepared ABTS^+^ free radical working solution, and the absorbance was measured at 734 nm and labeled AX. The results were expressed as% activity (%).


$$ABTS\mathrm{free}\mathrm{radical}\mathrm{scavenging}\left(\%\right)=\left(1-\frac{A\,x-{\text{A}}_{\text{x}\,0}}{A_{0}}\right)\times 100\%$$

Note: Ax0 is a 0.1 mL sample solution + 3.9 mL solvent; A0 is 0.1 mL solvent + 3.9 mL ABTS ^+^ working solution.

### 2.5. Mung bean polyphenol assay establishment

#### 2.5.1. Preparation of 20 polyphenol standards

The appropriate amount of the 20 standards were dissolved in dimethyl sulfoxide and the standard stock solution was configured at a concentration of 1 mg/mL. Next, 1 mL of each of the 20 standard stock solutions was removed and placed in a 100 mL volumetric flask. These stock solutions were then configured as a standard intermediate mixture of 10 μg/mL. This intermediate solution was further diluted into solutions with concentrations of 50, 100, 250, 500, 750, 1,000, and 1,500 ng/mL, respectively, after which the standard curves were plotted using the standard concentration and peak area as the horizontal and vertical axes, respectively. In this case, the method was established using a mixed standard solution of 1,000 ng/mL.

#### 2.5.2. Establishment of the method

Instrument platform: Q Exactive HF spectrometer; Column: aqueous. BEH C18, 1.7 μm/3.00 × 100 mm. Based on the non-targeted detection method established in a previous study, a mixture of 20 polyphenol standards was tested. Polyphenols were detected using full MS/dd-MS 2 (top-n) detection in positive/negative ion mode. Chromatographic conditions: mobile phase A for liquid chromatography comprised 1% formic acid in water, mobile phase B comprised acetonitrile, and the sample tray temperature was 4°C. Mass spectrometric conditions: electrospray voltage, 3.5 kV; sheath gas, 30 Arb; auxiliary gas, 20 Arb; ion transport tube temperature, 350°C; auxiliary gas heater temperature, 300°C; full mass spectrometric resolution, 120,000; MS/MS resolution, 60,000.

The scanning mode of the QE-HF-HRMS was changed to negative ionization (HEI-) and parallel reaction monitoring (PRM) modes after the detection of 20 specimens. The chromatographic conditions were maintained. The mass spectrometry conditions were as follows: electrospray voltage, 3.5 kV; sheath gas, 30 Arb; auxiliary gas, 20 Arb; ion transfer tube temperature, 350°C; auxiliary gas heater temperature, 300°C; MS/MS resolution ratio, 60,000. The target determination was performed according to the molecular weight of the polyphenols.

Finally, the main influencing factor collision energy (CE) is optimized. A simple optimization of the column temperature, flow Rate and mobile phase gradient was carried out, the rest is automatically optimized by the instrument.

### 2.6. Cell modeling methods

#### 2.6.1. Cell recovery and passaging methods

Caco-2 cell medium: DMEM + 10% FBS + 1% (penicillin-streptomycin solution). ModeK cell medium: DMEM + 10% FBS + 1% (penicillin-streptomycin solution). Resuscitate cells: Caco-2 and ModeK cells were removed from liquid nitrogen, and quickly placed in a 37°C water bath and shaken in a tube to gently dissolve the freezing solution; after dissolution, the cells were transferred to a centrifuge tube containing 5 ml of medium, centrifuged, the cells were collected, centrifuged at 1,000 r/min for 5 min at room temperature, and the supernatant was discarded. The cells were then suspended in complete medium containing 10% fetal bovine serum, inoculated into culture dishes, gently blown, and mixed, and incubated at 37°C with 5% CO_2_ saturation humidity.

##### 2.6.1.1. Cell passages

When the density of the cells reached 80%, the cells were passaged. The medium was discarded, and the cells were washed with PBS; 1–2 ml of 0.25% trypsin was added to digest the cells which were observed under a microscope. 1–2 min of digestion was seen when the cells were separated from each other and rounded, i.e., the digestion was complete; the trypsin was then quickly discarded, the complete medium was added, the cells were blown to make a single cell suspension, the cells were then passed at a ratio of 1:3, and the culture was expanded at 37°C and 5% CO_2_ saturated humidity.

#### 2.6.2. Cell model construction method

Caco-2 and mode-k cells were added to DMEM high sugar complete medium and placed in an incubator (37°C, 5%). Cell assays were performed when the cells were 80–90% fused. The control cells were incubated at 37°C with 5% CO_2_ concentration; the heat stress group was treated at 39°C, 41°C, and 43°C with 5% CO_2_ concentration for 2, 4, 6, and 8 h. The cells were first tested for cell morphology, cell viability, mRNA content of the HSP70 gene, and cellular glutathione peroxidase (GSH-Px) activity, lactate dehydrogenase (LDH) activity, total antioxidant activity, and total antioxidant activity. The time required for heat stress modeling was determined by changes in cell morphology, cell viability, HSP70 mRNA content, cellular GSH-Px, LDH activity, T-AOC, SOD, and MDA content.

#### 2.6.3. Cell viability MTT assay

Caco-2 and ModeK cells in the logarithmic growth phase were inoculated at 3 × 103 cells/well in a 96-well cell culture plate (16 plates) and incubated overnight at 37°C in a 5% CO_2_ incubator (100 μL sterile PBS was added to the wells around the cell wells); grouping: Group A, blank wells; Group B, modeK cells; and Group C, Caco-2 clls. Duration of action: incubate at 37°C, 39°C, 41°C, and 43°C for 2, 4, 6, and 8 h. Then, 10 μL MTT was added to each well and incubated at 37°C for 4 h. The medium was aspirated, 150 μL DMSO was added, and shaken for 10 min. The absorbance value of each well was then measured with the enzyme marker OD 570.

#### 2.6.4. Mitochondrial membrane potential and antioxidant index assay

The cells were cultured to the experimental state at different treatment temperatures or at the same treatment temperature for different treatment times. The assays were then performed according to the MDA, LDH, GSH-PX, T-AOC, SOD, and mitochondrial membrane potential assay kits.

#### 2.6.5. Changes in the relative mRNA expression of HSP27, HSP70, HSP90, Claudin-1, ZO-1, TNF-α, IL-1β, Bcl-2, and Bax genes

##### 2.6.5.1. Trizol method for RNA extraction

One milliliter of Trizol reagent was added to the cells, blown, mixed with a gun, transferred to a 1.5 ml RNase-free EP tube, and lysed for 10 min. 200 μL of chloroform was added, mixed several times *via* vigorous inversion, and left at room temperature for 5 min. The mixture was centrifuged for 15 min (4^°^C, 12,000 r/min), visible division into upper (RNA), middle (protein), and lower (DNA). The upper aqueous phase (approximately 400 μL) was transferred to a new 1.5 ml EP tube, 400 μL of isopropanol was added, mixed well, and left at room temperature for 10 min. The mixture was then centrifuged at 4°C, 12,000 r/min for 10 min, a white RNA precipitate was visible at the bottom of the tube. The supernatant was discarded, 1 ml of RNase-free 75% ethanol was added, vortexed, and centrifuged at 10,000 r/min for 5 min at 4°C. This was repeated only once. The supernatant was discarded and the RNA precipitate was dried in air for 5–10 min and dissolved in 20 μL of DEPC water. Two microliter of the dissolved RNA was then used to measure the OD260, OD280, and OD260/OD280 values using a microspectrophotometer to calculate the purity and concentration of RNA. The RNA mass was estimated according to the OD260/OD280 ratio, which was between 1.8 and 2.0, to meet experimental requirements. The concentration of sample RNA was calculated from the absorbance values according to the following formula:

Total RNA concentration (μg/μL) = OD260 × 40 × 10–3.
  The total RNA was stored in a refrigerator at −80°C for backup.
  The primer sequences, used for genetic testing (Table 1).

**TABLE 1:** Primer sequence list.

Gene	Primer	Sequence (5′-3′)	PCR products
b-actin	Forward	CACGATGGAGGGGCCGGA CTCATC	240 bp
	Reverse	TAAAGACCTCTATGCCAACACAGT	
Mus IL-1b	Forward	TCAGGCAGGCAGTATCACTC	250 bp
	Reverse	AGCTCATATGGGTCCGACAG	
Mus TNF-α	Forward	CGTCAGCCGATTTGCTATCT	206 bp
	Reverse	CGGACTCCGCAAAGTCTAAG	
Mus Bax	Forward	TTTTGCTACAGGGTTTCATCCA	181 bp
	Reverse	GTGTCCACGTCAGCAATCATC	
Mus Bcl2	Forward	AGCCCACCGTAACAATCAAG	147 bp
	Reverse	CCTGTCCCTTTGTCTTCAGC	
Mus ZO-1	Forward	CCAGCAACTTTCAGACCACC	154 bp
	Reverse	TTGTGTACGGCTTTGGTGTG	
Mus HSP70	Forward	GCAGACCTTCACCACCTACT	248 bp
	Reverse	CCTTGTCGTTGGTGATGGTG	
Mus HSP90	Forward	CTCCATGATCGGGCAGTTTG	239 bp
	Reverse	TCACCACTTCCTTGACCCTC	
Mus Claudin-1	Forward	GATGTGGATGGCTGTCATTG	246 bp
	Reverse	CGTGGTGTTGGGTAAGAGGT	
Mus HSP27	Forward	AGCGCTTCGGAGAAGATGT	150 bp
	Reverse	GGTCAGGAGGAGCAGGAAG	

#### 2.6.6. Analysis of significance

Univariate analysis of variance (ANOVA) and Duncan’s test (*p* < 0.05) using the SPSS 21 software were used for data calculation and significance analysis.

## 3. Results and analysis

### 3.1. Selection of mung bean components

In this study, total mung bean protein and mung bean globulin, which are the main storage proteins in mung beans, were selected. These two components represent the two types of crude protein and purified mung bean isolate proteins, respectively. Mung bean peptides also have good immunomodulatory activity as well as good antioxidant properties (25, 26), and the types of peptides chosen to mimic the digestion and absorption of the gastrointestinal tract after digestion were mung bean trypsin-hydrolyzed peptide and pepsin-hydrolyzed peptide, respectively. Polysaccharides also exhibit important physiological activities in organisms. Mung bean polysaccharides have been less studied, but differences have been found in the functional properties of mung bean polysaccharides obtained by different treatments. Mung bean polysaccharides obtained by aqueous extraction (solvent-free) and (hot) water-soluble polysaccharides exhibit antioxidant and immunomodulatory activities (27). Therefore, in this study, polysaccharides prepared by water extraction were chosen for the heat stress studies. Lipids supply energy and essential fatty acids to the body. Mung beans are low in lipids, including fat, phospholipids, and soy sterols. Polyphenols can be subdivided into crude extracts of mung bean polyphenols and monomeric polyphenols from mung beans. As heat stress can cause oxidative stress, *in vitro* antioxidant indicators were used in the prescreening process to screen for effective fractions of mung bean heat stress.

The polyphenols contained in mung beans from several origins were identified non-targeted by UHPLC-QE HF. The aim was to screen for polyphenolic substances contained simultaneously in mung beans from multiple origins. A total of 57 polyphenol fractions were screened as a result, (see Supplementary Table 1). There are currently 21 accurately detected polyphenols in mung beans (28–30), while mung bean polyphenols identified by non-targeted screening in this study were more abundant. Due to the large number of fractions, preliminary screening was carried out by reviewing the literature to select fractions with known heat stress modulating effects and polyphenolic substances without heat stress modulation-related studies, but with antioxidant properties. Fifteen polyphenolic substances were Caffeic acid, Ferulic Acid, Naringenin, Hesperetin, Quercetin, Chlorogenic acid, Curcumin, Puerain, Vitexin, Isovitexin, Naringin, Neohesperidin, Rutinum, Orientin, Gallic acid.

### 3.2. Determination of polyphenol content of mung bean monomers

The results from section “3.2 Determination of polyphenol content of mung bean monomers” show that the *in vitro* antioxidant properties of mung bean monomeric polyphenols are highly similar, but mung bean, as a daily dietary food for heat relief, should also have a higher content of fractions with heat stress-modulating effects. Therefore, the content of monomeric polyphenols in mung beans needs to be used as an indicator for the screening of major heat-stress-modulating polyphenol fractions. The 15 monomeric polyphenols screened in section “3.2 Determination of polyphenol content of mung bean monomers” were used as targets. Chlorogenic acid also contains five isomeric forms, cryptochlorogenic acid, neochlorogenic acid, isochlorogenic acid A, isochlorogenic acid B, and isochlorogenic acid C, were also added to the method development, a method for the detection of 20 polyphenols was developed based on UHPLC-QE HF.

The HRMS parameters of the 20 polyphenols were optimized by injecting QE-HF-HRMS with a continuous microflow jet pump in the PRM mode of negative ionization (HEI-). The detection results obtained a high sensitivity. In this study, the capillary voltage and characteristic fragment ions were fully optimized, and the carbon-equivalent (CE) values were 20, 30, 40, 50, and 60. The mass spectra are shown in Supplementary Figure 2. High response intensities for the parent ions of ferulic acid, naringenin, hesperetin, quercetin, and rutinum at CE = 20 and 30, and better results at CE = 40, 50, and 60, when the parent is not fully broken up and the target daughter ions have high response intensities. At CE = 20, 30, and 40, the parent ions of curcumin, orientin, cryptochlorogenic acid, neochlorogenic acid, and isochlorogenic acid B dissociated well, it CE > 40, the parent ion is completely broken up or there is too much fragmentation of the daughter ion. Gallic acid varied insignificantly at five collision energies, both of which were better. it CE = 30, 40, and 50, neohesperidin, puerarin, caffeic acid, ellagic acid, isochlorogenic acid A, and isochlorogenic acid C were dissociated with high corresponding intensity of the target daughter ions. It CE = 40 and 50, vitexin, and Isovitexin were most effective with high corresponding intensity of the target daughter ions. At CE = 30 and 40, naringenin dissociated best, with high parent ion response intensity and low daughter ion response intensity at CE < 30, and at CE > 40, the parent ion was completely broken up. Therefore, combining the effects of the 20 polyphenols, the optimized CE values were set to 30, 40, and 50. The mass spectrometric ion characteristics of the 20 mung bean polyphenols are shown in Table 2. The chromatograms of the 20 polyphenols under optimal conditions are shown in Supplementary Figure 4.

**TABLE 2:** Mass spectrometric signature ion list of 20 mung bean polyphenols.

	Name	Molecular formula	Molecular weight	Precursor ion	Retention time (min)	Characteristic ion 1	Characteristic ion 2
1	Caffeic acid	C_9_H_8_O_4_	180.16	179.03498	5.28	135.0441	107.0497
2	Ferulic acid	C_10_H_10_O_4_	194.18	193.05063	8.61	134.0363	178.0262
3	Naringenin	C_15_H_12_O_5_	272.25	271.0612	13.68	151.0026	119.0491
4	Hesperetin	C_16_H_14_O_6_	304.27	301.07176	14.31	164.0106	151.0027
5	Quercetin	C_15_H_10_O_7_	302.236	301.03538	12.60	151.0026	178.9976
6	Cryptochlorogenic acid	C_16_H_18_O_9_	354.31	353.08781	6.10	173.0446	135.0441
7	Chlorogenic acid	C_16_H_18_O_9_	354.31	353.08781	4.77	191.0553	161.0235
8	Neochlorogenic acid	C_16_H_18_O_9_	354.31	353.08781	2.11	191.0553	179.0341
9	Curcumin	C_21_H_20_O_6_	368.39	367.11871	12.61	301.0712	164.0106
10	Puerain	C_21_H_20_O_9_	416.378	415.10346	7.41	267.0657	295.0605
11	Vitexin	C_21_H_20_O_10_	432.378	431.09837	9.36	311.0555	283.0605
12	Isovitexin	C_21_H_20_O_10_	432.38	415.10346	9.36	311.0555	283.0605
13	Naringin	C_27_H_32_O_14_	580.535	579.17193	10.40	151.0026	271.0605
14	Neohesperidin	C_28_H_34_O_15_	610.56	609.18249	11.01	301.0712	151.0027
15	Rutinum	C_27_H_30_O_16_	610.52	609.1468	9.35	300.0269	271.0142
16	Isochlorogenic acid A	C_25_H_24_O_12_	516.453	515.1213	10.40	191.0552	179.0552
17	Isochlorogenic acid B	C_25_H_24_O_12_	516.453	515.0517	10.21	173.0447	191.0553
18	Isochlorogenic acid C	C_25_H_24_O_12_	516.45	515.1206	10.90	173.0446	135.0441
19	Orientin	C21H20O11	448.09	447.0932	8.74	327.0502	357.0607
20	Gallic acid	C7H6O5	170.01	169.01425	0.82	125.0233	169.0133

As the instrument is a combination of the parent ion selectivity of a high-performance quadrupole and the high-resolution accurate mass number (HR/AM) orbitrap detection technique, it exhibits excellent performance, and the MS conditions are crucial to the establishment of the method. Further, the LC conditions, including the flow rate, column temperature, and elution gradient, were also optimized. The flow rates were set as 0.3, 0.35, and 0.4 ml/min, it was observed that the higher the flow rate, the shorter the peak time, there was no significant change in the separation of the chromatographic peaks. Therefore, the flow rate was set as 0.4 ml/min. The column temperatures were set as 35°C, 40°C, and 45°C, The higher the temperature, the better the separation of the peaks. The column temperatures rate was set as 45°C. The mobile phase B was set to 28, 38, 48, and 58%, respectively. The best peak response intensities were found at 38 and 58%, with 38% being more stable, the Volume of mobile phase B es rate was set as 38%. The optimum chromatographic conditions are shown in Table 3. The chromatogram under optimal conditions is shown in Supplementary Figure 4. Qualitative and quantitative determination of 20 polyphenols in 18 min. The linearity results of the 20 polyphenol standard curves are shown in Supplementary Table 2.

**TABLE 3:** Optimal chromatographic conditions.

Time (min)	Flow (mL/min)	%B	Cure (μL)
0	0.4	5	5
3.5	0.4	5	5
15.5	0.4	38	5
17	0.4	38	5
17.1	0.4	5	5
18	0.4	5	5

**FIGURE 1:**
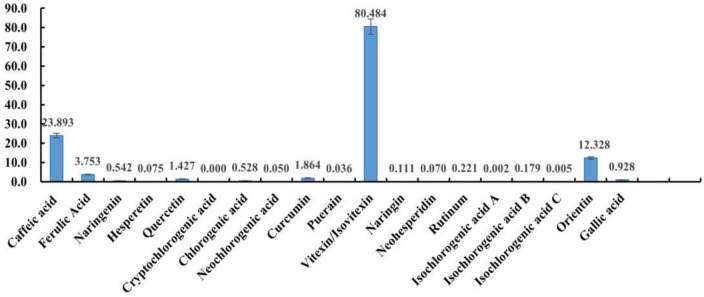
The content of 20 phenolics.

Detection of heat-stress-regulated polyphenolic fractions in mung beans was conducted using direct injection-mass spectrometry fingerprinting. After simple extraction, the samples were directly analyzed by mass spectrometry without excessive chromatographic separation. These 20 polyphenols can be detected and distinguished with great effectiveness and precise quantification. This method is a highly characteristic mass spectrometric fingerprinting technique. Among them, it was not possible to distinguish the two due to the mutual isomerization of vitexin and Isovitexin and the almost identical mass spectral fragments, the two isomers were also not separated in the chromatogram, which may be related to the column, and a chiral column should be chosen for the separation. But the mass spectra and chromatographic separation of the other components were very good. The method was then used to test the polyphenol content of mung beans and the results are shown in Figure 1 (The quantification results are the average of three parallel sample determinations). Polyphenolic substances with high content in mung beans are mucuna vitexin/Isovitexin, caffeic acid, and Orientin. Ferulic acid, Curcumin, quercetin, gallic acid, chlorogenic acid, and naringin are present in small amounts. The remaining components are present in very small amounts, Cryptochlorogenic acid is not present in mung beans. Vitexin/Isovitexin is a major polyphenol in mung beans. Caffeic acid was also detected. Individual studies have detected orientin in mung beans using non-targeted analysis (31), but there is no precise qualitative and quantitative method with corroboration, the present study confirms this. As shown by the DPPH radical scavenging rate of the monomeric polyphenols, the antioxidant properties of isovitexin are very poor. Therefore, from the point of view of content, vitexin, caffeic acid, and orientin were selected as mung bean monomeric polyphenol heat stress modulating components.

### 3.3. *In vitro* antioxidant activity of mung bean fractions

First, the antioxidant properties of each fraction of mung bean and 15 monomeric polyphenols were measured at high concentrations (10 mg/mL) using the DPPH scavenging rate as an indicator, the results of which are shown in Supplementary Figure 1A. Among the monomeric polyphenols, neohesperidin, hesperidin, naringenin, puerarin, isovitexin, and naringenin had relatively low DPPH clearance, so these were initially excluded. The total proteins, globulins, and polysaccharides in mung beans have lower antioxidant activity than most of the monomeric polyphenols, but given that they are nutritional fractions, subsequent experiments were continued. The antioxidant properties of the mung bean fractions were then compared based on the concentrations of DPPH and ABTS clearance at 50%. The results are shown in Supplementary Figure 1B. The scavenging rates of both free radicals increased with increasing concentration in the concentration range of each fraction, reaching over 90%. However, the strength of the antioxidant properties of the different fractions can be seen from the corresponding concentration range differences, with curcumin, orientin, rutin, gallic acid, quercetin, caffeic acid, ferulic acid, and chlorogenic acid in the range of 0.004–0.1 mg/mL. Vitexin in the range of 0.1–1.6 mg/mL. Among the mung bean fractions, the DPPH radical scavenging rate of mung bean polysaccharide, tryptase peptide, pepsin peptide, globulin, and total protein increased with increasing concentration. However, certain maximum values did not exceed 90%. The ABTS scavenging rate of the two mung bean proteins also increased with concentration, whereas the ABTS scavenging rate of the polysaccharide and the two mung bean peptides did not increase with concentration and tended to decrease slightly.

The scavenging rates of both free radicals from mung bean oil did not vary with concentration, and were relatively stable, ranging from 60 to 85%. Using a 50% scavenging rate of both free radicals as a criterion, combined with the range of scavenging rates of both free radicals, it can be seen that mung bean mixed polyphenols and monomeric polyphenols have good antioxidant properties, followed by oil and mung bean peptides, with proteins and polysaccharides having relatively poor antioxidant properties. Therefore, mixed polyphenols, oils, and two polypeptides were selected from the mung bean fraction for subsequent cellular experiments.

### 3.4. Screening of heat stress regulatory components in mung beans

The study first selected mode-K mouse small intestine epithelial cells for the construction of a heat stress model. Based on the results of the mode-K cell heat stress model screening, Caco-2 human colorectal adenocarcinoma cells were used to confirm the results of the mung bean fraction screening. Although many cellular heat stress models are available (32, 33), the conditions for heat stress construction differ for cells of different origins. Therefore, the conditions were based on the imbalance of oxidative stress Superoxide dismutase (SOD), GSH-PX, LDH, T-AOC, and MDA, loosening and “leakage” of intestinal epithelial junctions ZO-1 protein and Claudin protein, changes in intestinal inflammation, intestinal serum physicochemical parameters [Interleukin (IL-1β) and tumor necrosis factor (TNF-α)], apoptosis and changes in heat shock protein content due to heat stress-induced intestinal damage (HSP70, HSP27, HSP90). The above key indicators were used to construct and verify the cell model of heat stress. In this study, mild (39°C), moderate (41°C), and severe (43°C) heat stress models were constructed for both types of cells. For detailed results, see Supplementary Appendix—Experimental methods.

#### 3.4.1. Screening results of heat stress regulatory components of mung bean based on mode-k cell heat stress model

**FIGURE 2:**
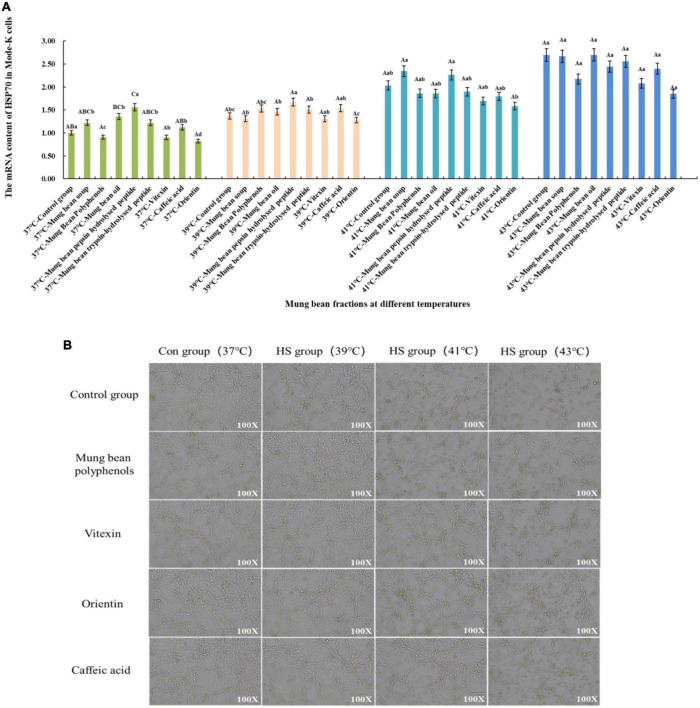
**(A)** Effect of mung bean components on HSP70 mRNA content in Mode-k cells. **(B)** Effect of key mung bean components on Mode-k cell morphology at different heat stress temperatures.

The effect of heat stress regulation investigated using the previously screened mung bean polyphenols, oils and fats, pepsin-hydrolyzed peptides, trypsin-hydrolyzed peptides, vitexin, orientin, and Caffeic acid using HSP70 mRNA content change as an indicator of heat stress. In addition, mung bean soup, a representative food in the daily diet to relieve heat, was compared with mung bean soup for heat stress regulation. The effect of each fraction on cell viability was determined by preliminary experiments, and a concentration of 30 μmol/L of each fraction was selected for the investigation of the regulatory effect of cellular heat stress. The results are shown in Figure 2A. When the components were co-cultured with cells at 37°C, mung bean polyphenols, vitexin, and orientin could decrease the HSP70 mRNA content, while the other components could increase the HSP70 mRNA content. Mung bean soup, vitexin and orientin decreased HSP70 mRNA content at 39°C, whereas other components increased HSP70 mRNA content. Mung bean polyphenols, mung bean oil, trypsin-hydrolyzed peptide, vitexin, orientin and caffeic acid decreased HSP70 mRNA content significantly at 41°C, whereas other components increased HSP70 mRNA content. Mung bean polyphenols, pepsin peptides, vitexin, orientin and caffeic acid decreased HSP70 mRNA content significantly at 43°C. The other components increased HSP70 mRNA content. The combined analysis revealed that mung bean polyphenols, vitexin, orientin, and caffeic acid almost all caused a downregulation of HSP70 mRNA content compared to the control group at each heat stress temperature. Although statistically not reaching the level of significant difference, the preventive and regulatory effects were better. Although green bean soup is useful in the daily diet to relieve heat stroke, a comparison shows that mung bean soup is not as effective as mixed and monomeric polyphenols in regulating cellular heat stress. Proteins, peptides, polysaccharides and lipids do not regulate HSP70 mRNA content well. These four substances are the components of mung beans screened for their heat stress regulatory group effects.

Figure 2B shows the effects of modulation of the cell morphology. The morphology of mode-k cells with the four components added at 37°C did not change, which initially indicated no significant toxicity to the cells, and the morphology of the control cells and cells with the four components added at 39°C under mild heat stress was not significantly different from that of the cells at 37°C. The cells under moderate heat stress at 41°C showed a shrinkage of cell morphology and a reduction in growth density compared to the control group, while the cell morphology of the cells with the four mung bean fractions improved significantly compared to the control group at 41°C. Cell morphology did not differ significantly from normal cells, and the effect of the three monomeric polyphenols was slightly better than that of mung bean polyphenols. Cells under severe heat stress at 43°C were significantly shrunken and oval in shape compared to those at 37°C. In contrast, the cells treated with the four mung bean fractions had significantly improved morphology and increased growth density. This indicates that the four mung bean fractions have significant effects on the regulation of cellular heat stress. Further, orientin has the best effect on the regulation of heat stress. All four fractions comprised polyphenols, which further indicates that polyphenols in mung beans are substances that regulate heat stress.

#### 3.4.2. Screening and verification results of heat stress regulatory components of mung bean based on Caco-2 cell heat stress model

**FIGURE 3:**
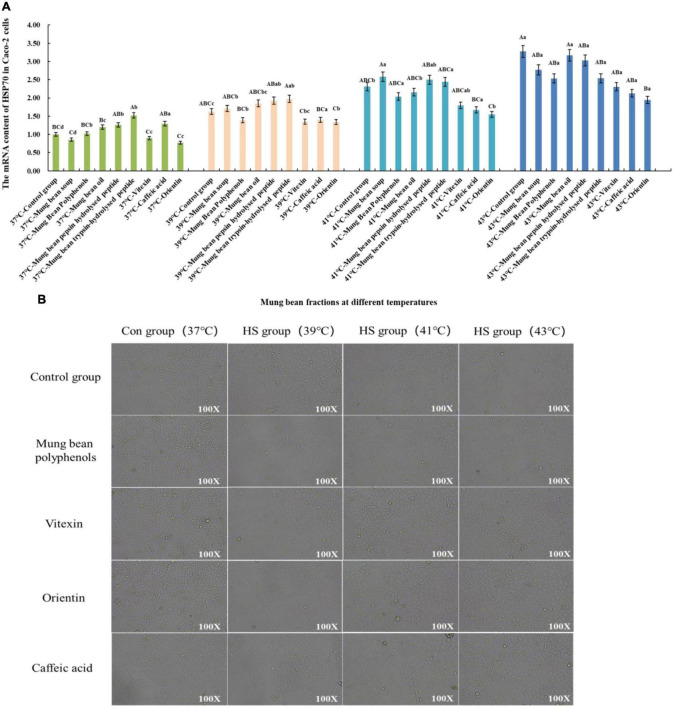
**(A)** Effect of mung bean components on HSP70 mRNA content in Caco-2 cells. **(B)** Effect of key mung bean components on Caco-2 cell morphology at different heat stress temperatures.

Based on the Caco-2 cell heat stress model, the heat stress regulatory components of mung bean were screened and verified by the change of HSP70 mRNA content. The results are shown in Figure 3A. Co-culture of the fractions with cells at 37°C showed that the mung bean polyphenols were at the same level as the control group. Mung bean broth, vitexin, and orientin decreased HSP70 mRNA content, while the other fractions increased HSP70 mRNA content. Mung bean polyphenols, vitexin, caffeic acid and orientin decreased HSP70 mRNA content, whereas other components increased HSP70 mRNA content at 39°C. At 41°C, mung bean polyphenols, mung bean oil, vitexin, orientin, and caffeic acid decreased the HSP70 mRNA content significantly, while the other components increased the HSP70 mRNA content. The HSP70 mRNA content of all fractions was lower than that of the control group at 43°C, however, mung bean polyphenols, vitexin, orientin, caffeic acid reduced the HSP70 mRNA content to a greater extent. The combined analysis revealed that mung bean polyphenols, vitexin, orientin, and caffeic acid almost all resulted in the downregulation of HSP70 mRNA content at each heat stress temperature compared to the control group. Although the degree of heat stress regulation did not reach statistical significance, its regulation improved. Among them, orientin was the most effective, with results identical to those of mode-k cells. The same can be found for mung bean soup is not as effective as mixed and monomeric polyphenols in regulating cellular heat stress. Proteins, peptides, polysaccharides and lipids do not regulate HSP70 mRNA content well. Confirming once again that these four substances were the fractions of mung beans screened for their heat stress regulatory group effects.

Figure 3B shows the cell morphology of the modulation effect. It can be clearly seen from the control group that the Caco-2 cells gradually shrink with the increase in heat stress temperature, the cell gap becomes larger, and the cell boundary becomes blurred. The morphology of the cells in the control group with the four fractions added at 37°C did not change, which tentatively indicated that there was no significant toxicity to Caco-2 cells. The morphology of the cells in the treatment group with the four fractions added at 39°C was similar to that of the cells at 37°C. Compared with the control group at 41°C, the cells with the four mung bean fractions showed less contraction of morphology and smaller cell gaps, and the regulation effect was better. The cell morphology was significantly improved by the addition of the four mung bean fractions compared with the control group at 43°C. Less cell shrinkage, clearer boundaries between cells, and increased cell growth density were observed. This indicated that the four mung bean fractions had a significant effect on the regulation of heat stress in Caco-2 cells. The effect of the four monomeric polyphenols was slightly better than that of mung bean polyphenols in terms of cell morphology.

The mung bean fractions screened by the mode-k cell heat stress model were validated in the Caco-2 cell heat stress model by combining the results of all indicators under six heat stress models for both cells. Very few studies have been conducted on the regulation of heat stress by mung beans and their foods. Cao et al. (8) found that vitexin and isovitexin were the main antioxidant components of mung beans (more than 96% of which was present in the seed coat of the beans), both of which could be absorbed into rat plasma through gavage. The plasma levels of MDA and the activities of LDH and nitric oxide synthase (NOS) were significantly reduced in rats fed mung bean peel extract before and after heat stress, whereas the levels of T-AOC and GSH-Px (a quantitative assessment of oxidative stress) were significantly increased. It was confirmed that mung bean flavonoids could alleviate heat stress in rats by modulating the antioxidant levels in the body. In this study, the heat stress-modulating effects of the screened mung bean fractions were investigated at the cellular level. In addition to vitexin, orientin, and caffeic acid have been found to be effective in heat stress regulation. Orientin is a flavonoid, caffeic acid are phenolic acid compounds. This suggests that phenolic acid compounds also have heat stress-modulating effects. The results of this study were similar to those reported by Cao et al. Once again, it was confirmed that mung bean polyphenols are a major substance in their heat stress-modulating effect and are closely related to the antioxidant properties of polyphenols.

Orientin has been shown to have anti-inflammatory, antibacterial, antiviral, and antioxidant properties (34–36). Clinical studies have shown that Orientin is a potential treatment for Alzheimer’s disease (37), as well as an anti-tumor agent (38). However, studies on the Orientin of heat stress by herbicides have not been reported. Polyphenols, as secondary metabolites, are also a source of feedback from plants to adapt to environmental changes. Wang et al. (39) found that short-term heat stress in plants resulted in a significant increase in caffeic acid content, suggesting a potential role for caffeic acid as an emergent signaling molecule. Bhardwaj Rachana and Ramandeep (40) study confirmed that protective Role of Pre-treatment with Caffeic acid in Wheat Seedlings Against Heat Stress Induced Oxidative Damage. Caffeic acid has a heat stress resistance effect, and its antioxidant properties have been extensively demonstrated (41). However, caffeic acid has not been reported at the cellular or animal level in response to heat stress.

The results showed that the modeling time for both cell models was 6 h. The modeling time in the existing heat stress cell models was also variable, ranging from 1, 2, 4, to 6 h, which was closely related to the cell source and type. However, many of the available reports did not provide a clear explanation of the basis for the choice of modeling time or only used cell viability as an indicator. There was also some variation in the choice of heat stress temperature, ranging from 38 to 43°C. Many reports have only selected a single heat stress temperature for the study, and the temperature settings for different levels of heat stress have not been adequately studied. The results of this study also confirmed that 39°C, 41°C, and 43°C had different levels of heat stress and that statistically significant differences existed in all indicators at all three temperatures.

#### 3.4.3. Confirmation of the main heat stress components

**FIGURE 4:**
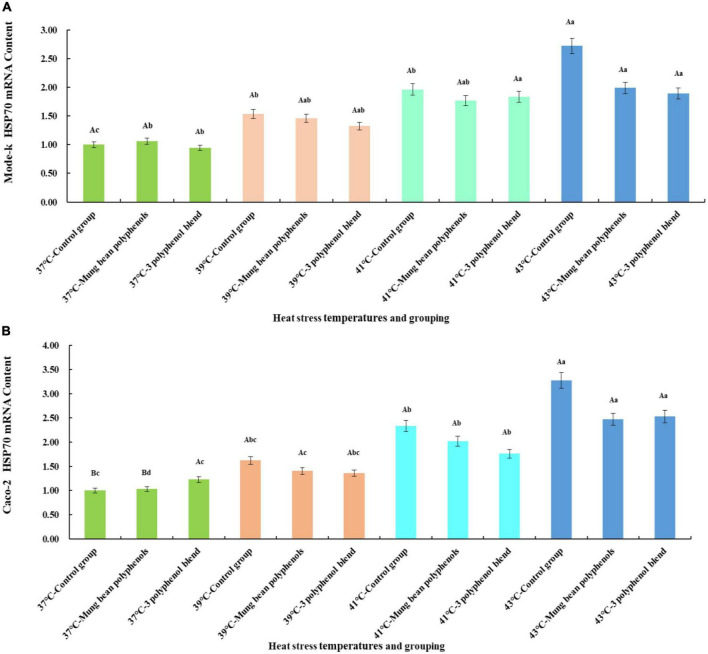
**(A)** Results of the regulation of HSP70 mRNA content in Mode-k cells by mung bean polyphenols and a mixture of three monomeric mung bean polyphenols. **(B)** Results of the regulation of HSP70 mRNA content in Caco-2 cells by mung bean polyphenols and a mixture of three monomeric mung bean polyphenols.

From the quantitative results, it is clear that a high proportion of vitexin, orientin, and caffeic acid was screened. Therefore, to confirm whether the three monomeric polyphenols might be the main components of mung bean that regulate heat stress, the three monomeric polyphenols were formulated in proportion to their content. The HSP70 mRNA content was then compared with that of mung bean polyphenols. The results are shown in Figure 4.

The mode-k cell and Caco-2 cell heat stress model is shown in Figure 4. A significant increase in HSP70 mRNA levels occurred in all three control groups at heat stress temperatures compared to the 37°C control group, indicating that the two types of cells heat stress model was valid. Mung bean polyphenols at 37°C slightly increased HSP70 mRNA content in mode-k cells, and a mixture of three monomeric polyphenols slightly decreased HSP70 mRNA content, but the differences between them were not significant. The mixture of mung bean polyphenols and three monomeric polyphenols reduced HSP70 mRNA content in mode-k and Caco-2 cells at all three heat stress temperatures, and the reduction was greater at higher heat stress temperatures but did not reach a significant reduction. A comparison of the regulatory effect of mung bean polyphenols with that of the three monomeric polyphenols showed that they are similar, with a mixture of the three monomeric polyphenols being somewhat more effective. The results were the same for both cell types, indicating that the mixture of the three monomeric polyphenols was similarly or better regulated than the mung bean polyphenols. The mung bean polyphenols contained other classes of polyphenols in addition to the three monomeric polyphenols. However, the mixture of the three monomeric polyphenols was more effective than the mung bean polyphenols, suggesting that the three monomeric polyphenols are likely to be the main components of heat stress regulation in mung beans. These results reaffirm that mung bean polyphenols have a heat stress-modulating effect.

## 4. Conclusion

The study was based on the mode-k mouse intestinal epithelial cell heat stress model and Caco-2 human rectal colon cancer cells, and six heat stress cell models were successfully constructed at 39°C (mild), 41°C (moderate), and 43°C (severe) using a combination of real-time quantitative PCR and physicochemical index assays. The main nutrient fractions and polyphenolic substances in mung beans were screened for heat stress regulatory fractions, using HSP70 as a key indicator. The results confirmed that polyphenols were the main heat stress-regulating components in mung beans, among which orientin, vitexin, and caffeic acid were likely the main components. The higher the degree of heat stress, the more significant the regulation effect. Both flavonoids and phenolic acids in mung beans have heat stress-modulating effects. The heat-stress-modulating effects of polyphenols are closely related to their antioxidant properties. A qualitative and quantitative assay for 20 polyphenols was established using targeted analysis. The results of this study provide theoretical support for the regulation of heat stress by polyphenols. However, the present study was a preliminary screening, and the components in mung bean were diverse, with interactions among them. Whether the complexes of components in mung beans have heat stress-modulating effects or other effects requires further investigation. The mechanism of heat stress regulation by mung bean polyphenolic substances at the cellular and *in vivo* levels in animals was the next step in the research program.

## Data availability statement

The data presented in the study are deposited in the Figshare repository, accession number https://figshare.com/s/7b59de689f7699d41a22.

## Author contributions

YF: writing—original draft, methodology, and visualization. XF: conceptualization and writing—review and editing. SZ and DS: formal analysis and methodology. YM: data curation and visualization. HW: data curation and investigation. XG: formal analysis and investigation. CW and HY: supervision, project administration, and funding acquisition. All authors contributed to the article and approved the submitted version.

## References

[1] Pawar SS Sajjanar B Lonkar VD Nitin KP Kadam AS Nirmale AV Assessing and mitigating the impact of heat stress in poultry. *Adv Anim Vet Sci.* (2016) 4:332–41. 10.14737/journal.aavs/2016/4.6.332.341

[2] Wouters H De Ridder K Poelmans L Willems P Brouwers J Hosseinzadehtalaei P Heat stress increase under climate change twice as large in cities as in rural areas: a study for a densely populated midlatitude maritime region. *Geophys Res Lett.* (2017) 44:8997–9007. 10.1002/2017gl074889

[3] Lemonsu A Viguié V Daniel M Masson V. Vulnerability to heat waves: impact of urban expansion scenarios on urban heat island and heat stress in Paris (France). *Urban Clim.* (2015) 14:586–605. 10.1016/j.uclim.2015.10.007

[4] Salata F Golasi I Petitti D de Lieto Vollaro E Coppi M de Lieto Vollaro A. Relating microclimate, human thermal comfort and health during heat waves: an analysis of heat Island mitigation strategies through a case study in an urban outdoor environment. *Sustain Cities Soc.* (2017) 30:79–96. 10.1016/j.scs.2017.01.006

[5] Caulfield MP Cambridge H Foster SF McGreevy PD. Heat stress: a major contributor to poor animal welfare associated with long-haul live export voyages. *Vet J.* (2014) 199:223–8. 10.1016/j.tvjl.2013.09.018 24157340

[6] Ellamie AM Fouda WA Ibrahim WM Ramadan G. Dietary supplementation of brown seaweed (*Sargassum latifolium*) alleviates the environmental heat stress-induced toxicity in male *Barki sheep* (*Ovis aries*). *J Therm Biol.* (2020) 89:102561. 10.1016/j.jtherbio.2020.102561 32364993

[7] Watts N Adger WN Agnolucci P Blackstock J Byass P Cai W Health and climate change: policy responses to protect public health. *Lancet.* (2015) 386:1861–914. 10.1016/s0140-6736(15)60854-6 26111439

[8] Cao D Li H Yi J Zhang J Che H Cao J Antioxidant properties of the *Mung bean* flavonoids on alleviating heat stress. *PLoS One.* (2011) 6:e21071. 10.1371/journal.pone.0021071 21695166 PMC3112222

[9] Lord-Fontaine S Averill-Bates DA. Heat shock inactivates cellular antioxidant defenses against hydrogen peroxide: protection by glucose. *Free Radic Biol Med.* (2002) 32:752–65. 10.1016/s0891-5849(02)00769-4 11937301

[10] Altan Ö Pabuçcuoğlu A Altan A Konyalioğlu S Bayraktar H. Effect of heat stress on oxidative stress, lipid peroxidation and some stress parameters in broilers. *Br Poult Sci.* (2003) 44:545–50. 10.1080/00071660310001618334 14584844

[11] Mujahid A Yoshiki Y Akiba Y Toyomizu M. Superoxide radical production in chicken skeletal muscle induced by acute heat stress. *Poult Sci.* (2005) 84:307–14. 10.1093/ps/84.2.307 15742968

[12] Lin H Decuypere E Buyse J. Acute heat stress induces oxidative stress in broiler chickens. *Comp Biochem Physiol Part A.* (2006) 144:11–7. 10.1016/j.cbpa.2006.01.032 16517194

[13] Sakakibara H Shimoi K. Anti-stress effects of polyphenols: animal models and human trials. *Food Funct.* (2020) 11:5702–17. 10.1039/d0fo01129k 32633737

[14] Peng X Zheng Z Cheng KW Shan F Ren GX Chen F Inhibitory effect of *Mung bean* extract and its constituents vitexin and isovitexin on the formation of advanced glycation endproducts. *Food Chem.* (2008) 106:475–81. 10.1016/j.foodchem.2007.06.016

[15] Slawinska A Mendes S Dunislawska A Siwek M Zampiga M Sirri F Avian model to mitigate gut-derived immune response and oxidative stress during heat. *Biosystems.* (2019) 178:10–5. 10.1016/j.biosystems.2019.01.007 30659866

[16] Gilles C Helen B Hella H Sue C. The *Drosophila* Dpit47 protein is a nuclear Hsp90 co-chaperone that interacts with DNA polymerase alpha. *J Cell Sci.* (2001) 114:2015–25. 10.1242/jcs.114.11.2015 11493638

[17] Beckham JT Wilmink GJ Mackanos MA Takahashi K Contag CH Takahashi T Role of HSP70 in cellular thermotolerance. *Lasers Surg Med.* (2008) 40:704–15. 19065555 10.1002/lsm.20713

[18] Du M Xie J Gong B Xu X Tang W Li X Extraction, physicochemical characteristics and functional properties of *Mung bean* protein. *Food Hydrocoll.* (2018) 76:131–40. 10.1016/j.foodhyd.2017.01.003

[19] Zhang S Sheng Y Feng Y Diao J Wang C Zhang D. Changes in structural and functional properties of globulin–polyphenol complexes in *Mung beans*: exploration under different interaction ratios and heat treatment conditions. *Int J Food Sci Technol.* (2021) 57:1–34. 10.1111/ijfs.15180

[20] Ye HZ. *Preparation, separation, purification and structural identification of Mung bean antioxidant peptides.* Nanchang: Nanchang University (2021). p. 14–25.

[21] Song QQ. *Research on physicochemical, antioxidant properties of Mung bean polysaccharides activity and its effects on intestinal health of mice.* Nanchang: Nanchang University (2020). p. 12–3.

[22] Dou KN Bai CQ. Extraction process optimization and fatty acid composition analysis of glycyrrhiza oil. *J Chin Cereals Oils Assoc.* (2018) 33: 102–6.

[23] Zhang S. *Study on the processing methods on the functional properties and their interaction of polyphenols and proteins in Mung bean.* Heilongjiang: Heilongjiang Bayi Agricultural University (2021). p. 17–8.

[24] Lahouar L El Arem A Ghrairi F Chahdoura H Ben Salem H El Felah M Phytochemical content and antioxidant properties of diverse varieties of whole barley (*Hordeum vulgare* L.) grown in Tunisia. *Food Chem.* (2014) 145:578–83. 10.1016/j.foodchem.2013.08.102 24128517

[25] Li M Zhang Y Xia S Ding X. Finding and isolation of novel peptides with anti-proliferation ability of hepatocellular carcinoma cells from *Mung bean* protein hydrolysates. *J Funct Foods.* (2019) 62:103557. 10.1016/j.jff.2019.103557

[26] Xie J Du M Shen M Wu T Lin L. Physico-chemical properties, antioxidant activities and angiotensin-I converting enzyme inhibitory of protein hydrolysates from *Mung bean* (*Vigna radiate*). *Food Chem.* (2019) 270:243–50. 10.1016/j.foodchem.2018.07.103 30174041

[27] Yao Y Zhu Y Ren G. Immunoregulatory activities of polysaccharides from *Mung bean*. *Carbohydr Polym.* (2016) 139:61–6. 10.1016/j.carbpol.2015.12.001 26794947

[28] Yao Y Yang X Tian J Liu C Cheng X Ren G. Antioxidant and antidiabetic activities of black *Mung bean* (*Vigna radiata* L.). *J Agric Food Chem.* (2013) 61:8104–9. 10.1021/jf401812z 23947804

[29] Paja̧k P Socha R Gałkowska D Rożnowski J Fortuna T. Phenolic profile and antioxidant activity in selected seeds and sprouts. *Food Chem.* (2014) 143:300–6. 10.1016/j.foodchem.2013.07.064 24054243

[30] Bai Y Zhang Q Wang B Zhang M Xu Y Li S Plasma pharmacokinetics, bioavailability, and tissue distribution of four c-glycosyl flavones from *Mung bean* (*Vigna radiata* L.) seed extracts in rat by ultrahigh-performance liquid chromatography–tandem mass spectrometry. *J Agric Food Chem.* (2017) 65:5570–80. 10.1021/acs.jafc.7b02053 28627167

[31] Xiao ZM. *The inhibitory effect of orientin in Mung bean on liver cancer and the effect of processing mode on its activity.* Heilongjiang: Heilongjiang Bayi Agricultural University (2020). p. 30–1.

[32] Pan ZG He X Shao Y Chen WD Fang BJ. ROS/JNK-mediated lysosomal injury in rat intestinal epithelial-6 cells during heat stress. *J Therm Biol.* (2022) 109:103326. 10.1016/j.jtherbio.2022.103326 36195392

[33] Hooper HB dos Santos Silva P de Oliveira SA Merighe GKF Negrão JA. Acute heat stress induces changes in physiological and cellular responses in *Saanen goats*. *Int J Biometeorol.* (2018) 62:2257–65. 10.1007/s00484-018-1630-3 30368674

[34] Du SK Yu XZ Li ZX. *In vitro* antioxidative activity of ethanol extracts of food legume. *J Chin Inst Food Sci Technol.* (2012) 12:14–9.

[35] Liang SL Liang Q Zhong WH Li QY Yan FG Zhou XG. Anti-inflammatory and analgesic effects of *Polygonum* orientale extract. *Chin Tradit Herb Drugs.* (2014) 45:3131–5.

[36] Xiao Q Qu Z Zhao Y Yang L Gao P. Orientin ameliorates LPS-induced inflammatory responses through the inhibitory of the NF-κB pathway and NLRP3 inflammasome. *Evid Based Complement Altern Med.* (2017) 2017:2495496. 10.1155/2017/2495496 28197210 PMC5288532

[37] Zhong Y Zheng Q Sun C Zhang Z Han K Jia N. Orientin improves cognition by enhancing autophagosome clearance in an Alzheimer’s mouse model. *J Mol Neurosci.* (2019) 69:246–53. 10.1007/s12031-019-01353-5 31243684

[38] Fang AN Wang SH Tian QQ Zhu DX. Effects of orientin and vitexin from *Trollius* chinensis on the growth and apoptosis of esophageal cancer EC-109 cells. *Oncol Lett.* (2015) 10:2627–33. 10.3892/ol.2015.3618 26622901 PMC4580036

[39] Wang J Yuan B Huang B. Differential heat-induced changes in phenolic acids associated with genotypic variations in heat tolerance for hard fescue. *Crop Sci.* (2019) 59:667. 10.2135/cropsci2018.01.0063

[40] Bhardwaj Rachana D Ramandeep K. Protective role of pre-treatment with different phenolic acids in wheat seedlings against heat stress induced oxidative damage. *Indian J Agric Biochem.* (2017) 30:147–55. 10.5958/0974-4479.2017.00024.7

[41] Khan FA Maalik A Murtaza G. Inhibitory mechanism against oxidative stress of caffeic acid. *J Food Drug Anal.* (2016) 24:695–702. 10.1016/j.jfda.2016.05.003 28911606 PMC9337298

[42] Ali NM Mohd Yusof H Yeap SK Ho WY Beh BK Long K Anti-inflammatory and antinociceptive activities of untreated, germinated, and fermented *Mung bean* aqueous extract. *Evid Based Complement Altern Med.* (2014) 2014:350507. 10.1155/2014/350507 25045389 PMC4089844

[43] Chai WM Wei QM Deng WL Zheng YL Chen XY Huang Q Anti-melanogenesis properties of condensed tannins from *Vigna* angularis seeds with potent antioxidant and DNA damage protection activities. *Food Funct.* (2019) 10:99–111. 10.1039/c8fo01979g 30565612

[44] Das A. Heat stress-induced hepatotoxicity and its prevention by resveratrol in rats. *Toxicol Mech Methods.* (2011) 21:393–9. 10.3109/15376516.2010.550016 21426263

[45] Frijhoff J Winyard PG Zarkovic N Davies SS Stocker R Cheng D Clinical relevance of biomarkers of oxidative stress. *Antioxid Redox Signal.* (2015) 23:1144–70. 10.1089/ars.2015.6317 26415143 PMC4657513

[46] Giusti F Caprioli G Ricciutelli M Torregiani E Vittori S Sagratini G. Analysis of 17 polyphenolic compounds in organic and conventional legumes by high-performance liquid chromatography-diode array detection (HPLC-DAD) and evaluation of their antioxidant activity. *Int J Food Sci Nutr.* (2017) 69:557–65. 10.1080/09637486.2017.1399258 29117733

[47] Gupta N Srivastava N Bhagyawant SS. Vicilin—A major storage protein of mungbean exhibits antioxidative potential, antiproliferative effects and ACE inhibitory activity. *PLoS One.* (2018) 13:e0191265. 10.1371/journal.pone.0191265 29408872 PMC5800569

[48] He S Li S Arowolo MA Yu Q Chen F Hu R Effect of resveratrol on growth performance, rectal temperature and serum parameters of yellow-feather broilers under heat stress. *Anim Sci J.* (2019) 90:401–11. 10.1111/asj.13161 30623539

[49] Ketha K Gudipati M. Immunomodulatory activity of non starch polysaccharides isolated from green gram (*Vigna radiata*). *Food Res Int.* (2018) 113:269–76. 10.1016/j.foodres.2018.07.010 30195521

[50] Li X Yang Y Liu S Yang J Chen C Sun Z. Grape seed extract supplementation attenuates the heat stress-induced responses of jejunum epithelial cells in *Simmental* × qinchuan steers. *Br J Nutr.* (2014) 112:347–57. 10.1017/s0007114514001032 24846452

[51] Liyanage R Kiramage C Visvanathan R Jayathilake C Weththasinghe P Bangamuwage R Hypolipidemic and hypoglycemic potential of raw, boiled, and sprouted *Mung beans* (*Vigna radiata* L. Wilczek) in rats. *J Food Biochem.* (2017) 42:e12457. 10.1111/jfbc.12457

[52] Lopes L Martins M Farias L Brito A Lima G Carvalho V Cholesterol-lowering and liver-protective effects of cooked and germinated *Mung Beans* (*Vigna radiata* L.). *Nutrients.* (2018) 10:821. 10.3390/nu10070821 29949855 PMC6073478

[53] Nakatani A Li X Miyamoto J Igarashi M Watanabe H Sutou A Dietary *Mung bean* protein reduces high-fat diet-induced weight gain by modulating host bile acid metabolism in a gut microbiota-dependent manner. *Biochem Biophys Res Commun.* (2018) 501:955–61. 10.1016/j.bbrc.2018.05.090 29777704

[54] Sordillo LM Aitken SL. Impact of oxidative stress on the health and immune function of dairy cattle. *Vet Immunol Immunopathol.* (2009) 128:104–9. 10.1016/j.vetimm.2008.10.305 19027173

[55] Sahin K Orhan C Tuzcu M Ali S Sahin N Hayirli A. Epigallocatechin-3-gallate prevents lipid peroxidation and enhances antioxidant defense system via modulating hepatic nuclear transcription factors in heat-stressed quails. *Poult Sci.* (2010) 89:2251–8. 10.3382/ps.2010-00749 20852116

[56] Sonklin C Laohakunjit N Kerdchoechuen O. Assessment of antioxidant properties of membrane ultrafiltration peptides from *Mungbean* meal protein hydrolysates. *PeerJ.* (2018) 6:e5337. 10.7717/peerj.5337 30065890 PMC6065462

[57] Vandana GD Bagath M Sejian V Krishnan G Beena V Bhatta R. Summer season induced heat stress impact on the expression patterns of different toll-like receptor genes in *Malabari goats*. *Biol Rhythm Res.* (2018) 50:466–82. 10.1080/09291016.2018.1464638

[58] Watanabe H Inaba Y Kimura K Asahara S Kido Y Matsumoto M Dietary *Mung bean* protein reduces hepatic steatosis, fibrosis, and inflammation in male mice with diet-induced, nonalcoholic fatty liver disease. *J Nutr.* (2016) 147:52–60. 10.3945/jn.116.231662 27903831

[59] Xie J Ye H Du M Yu Q Chen Y Shen M. *Mung bean* protein hydrolysates protect mouse liver cell line Nctc-1469 cell from hydrogen peroxide-induced cell injury. *Foods.* (2019) 9:14. 10.3390/foods9010014 31877918 PMC7023459

[60] Zhu YG. Technical measures to alleviate heat stress in pigs. *China Anim Health.* (2021) 23:79. 10.3969/j.issn.1008-4754.2021.04.05

