# Supplementary material for: Screening of heat stress-regulating active fractions in mung beans
Source: Front Nutr. 2023 Feb 20;9:1102752. doi: 10.3389/fnut.2022.1102752 (PMC9986443; doi:10.3389/fnut.2022.1102752)
Supplement: Supplementary file 1 [file Presentation_1.pdf]

Appendix - Experimental methods

1. Mung bean fraction extraction methods

1.1 Mung bean protein extraction

Globulin: defatted mung bean flour (10.0%, w/v) was added to an acidified salt solution (containing 0.025 mol/L hydrochloric acid at pH 3.5) at a concentration of 0.5 mol/L and stirred for 2 h at room temperature, maintaining a stable pH. The suspension was centrifuged at 12 000 x g for 20 min in a cryogenic freezing centrifuge, the supernatant was diluted with three times the volume of distilled water and left to stand in an ice-water bath for 30 min, and the precipitate was collected by centrifugation at 4 °C (12 000 x g, 20 min) and repeated twice. The sample was dialyzed in distilled water at 4 °C for 48 h and then lyophilized to obtain a globulin-rich mung bean protein sample（Zhang et al., 2021）.

Total protein: We implemented the method of Du et al. (2018) with slight modifications. Mung beans were peeled, crushed, sieved, defatted with hexane, and mixed with distilled water at a solid-liquid ratio of 1:10. The pH was adjusted to 9.0 with 2 mol/L NaOH solution, extracted with stirring for 50 min, and centrifuged at 4500 r/min for 20 min. The supernatant was transferred to a beaker and the precipitate was mixed at a solid-liquid ratio of 1:10. The supernatant was centrifuged at 4 500 r/min for 20 min. The precipitate was freeze-dried and stored at -20 °C until use.

1.2 Mung bean peptide extraction method

A 3% mung bean protein solution with substrate concentration was prepared, pre-cooked in boiling water bath for 15 min, the temperature was adjusted to 37 °C, the pH was adjusted to the optimum enzymatic pH of protease (pepsin pH 2, trypsin pH 8) with 1 mol/L NaOH or HCl solution. Then, 2% protease ([E]/[S]) was added and the digestion was stirred in a constant-temperature water bath, after which 1 mol/L NaOH or HCl was added to maintain a constant pH during digestion. After 3 h of continuous digestion, the pH of the digestion solution was adjusted to 7.0, and the temperature was rapidly increased to 95 ℃. The heat was maintained to inactivate the enzyme for 10-15 min. After cooling to room temperature, the peptide was centrifuged at 5000 rpm for 30 min, and the supernatant comprised mung bean peptide. The supernatant was then centrifuged at 5000 r/min for 30 min. The mung bean peptide solution was freeze-dried in a vacuum to produce mung bean peptide powder (Ye, 2021).

1.3 Mung bean polysaccharide extraction method

First, 600 g of defatted mung bean flour was soaked in 4800 mL of ethanol solution (80%, v/v) for 24 h to remove pigments and fat and then dried to remove the alcohol. The dried residue was extracted twice with hot water (residue:water = 1:20) for 2 h. The resulting solution was filtered and concentrated to obtain an ethanol concentration of 80% (v/v) by adding 95% (v/v) ethanol to the mixture and to obtain the final precipitate. After the solution was centrifuged, redissolved, and concentrated to 1/3 of the original solution volume, the proteins were removed with papain (3 g/L) after which the solution was dialyzed, concentrated, and lyophilized to obtain mung bean whole bean polysaccharide powder (Song, 2020).

1.4 Mung bean oil extraction method

Five grams of mung bean powder was weighed into a round-bottom flask of a Soxhlet extractor and a certain amount of petroleum ether was added. The Soxhlet extractor was mounted on a constant-temperature water bath, heated, and condensed to reflux to extract mung bean oil. After extraction, the cooled extract was transferred to a rotary evaporator to recover the solvent by distillation, and the concentrate was dried in a constant-temperature blast dryer to a constant mass to obtain mung bean oil. Each experiment was performed in parallel three times (Dou & Bai, 2018).

2. RT reverse transcription to cDNA

Reverse transcription reaction system: (two-step method)

Table 1 Reverse transcription reaction system-1

RT1: Genomic DNA removal 42℃ 2 min

Reagent	Volume（μl）
Template RNA	2μg
4 × gDNA wiper Mix	4
Oligo (dT)23VN primer (10 μM)	1
RNase-free ddH2O	Up to 16

RT2: Preparing the reverse transcription reaction system

Reagent	Volume（μl）
5 × HiScript II Select qRT SuperMix II	4
Reaction solution for step 1	16

Reaction conditions:

Temperature	Time
50℃	15min
85℃	5s
4℃	10min

Real-time fluorescence quantitative PCR assay

Reaction system.

Table 2 Real-time fluorescent quantitative PCR reaction system

cDNA	4μl
Forward Primer (10μM)	0.4μl
Reverse Primer (10μM)	0.4μl
SYBR Green Master Mix	10μl
50×ROX Reference Dye 2	0.4μl
H2O	4.8μl

Reaction procedure:

Project	Temperature	Time	Number of cycles
Pre-denaturing	95℃	10min	1
Denaturation	95℃	15sec	40
Annealing extension	60℃	60sec	
	95℃	15sec	
Melting curve acquisition	60℃	60sec	1
	95℃	15sec	

Replicate wells: 3
cDNA dilution: 5

3. Results of heat stress model construction index for cells

3.1 Mode-k cell model construction and heat stress component screening

As shown in Figure A1(a), normally cultured mode-k cells at 37 °C showed an irregular shape with wide middle and elongated sides. Simultaneous with heat stress, the elongated shape of the two sides of the cells gradually decreased with increasing temperature and finally became oval in shape. Overall, the cells changed from a patchy state to a scattered distribution. At 39 °C, the cell morphology did not differ much from that at 37 °C. At 41 °C, the moderate heat stress level changed significantly compared with the normal temperature of 37 °C. At 43 °C, the cell condition became significantly worse. The cell shrinkage was more severe. At the same treatment temperature, the cell morphology also changed from elongated to oval-like as the duration of heat stress increased, and with longer heat stress, the morphological changes were more significant. The effect of heat stress on cell morphology was significant and increased with an increase in the degree of heat stress and duration of heat stress.

Figure A1(b) shows the results of the MTT assay on mode-K cells. Changes in cell activity at different heat stress temperatures and heat stress times at the same temperature can be observed. ABCD in the Figure 4(b) represents a significant ($p < 0.05$) change within the group, and abcd represents a significant ($p < 0.05$) change between the groups. As can be seen from Figure 4, the cell viability at 37 °C was good and the cell viability of the 39 °C treated group showed a process of decreasing and then increasing back. The overall cell viability was not significantly different from that at 37 °C. The overall cell viability decreased with increasing heat stress at 41 °C, non-significantly at 2 h, 4 h and 6 h, and significantly at 8 h. The overall cell viability decreased with increasing heat stress at 43 °C, non-significantly at 2 h, 4 h, and significantly at 6 h and 8 h. In the different heat stress temperature comparison groups, cell viability showed a significant decrease after 6 h of treatment at 41 °C compared to 37 °C and 39 °C. At 43 °C was significantly different from the other temperatures at After 4 h of heat stress. When the heat stress temperature was different, there was no significant change in cell viability at each heat stress temperature after 2 h of heat stress. Significant differences existed between 41 °C and 43 °C at 6 h and 8 h of heat stress, and cell viability was not significantly different between 39 °C and 37 °C but was reduced. It was also confirmed that the three heat-stress temperatures could represent different levels of heat stress.

The expression of HSP70 is induced at high levels in the body in the presence of heat shock, and HSP70 directly senses changes in the intra- and extracellular environments. Therefore, the expression of HSP70 can be used as a biological marker of heat stress in response to heat stimulation, which induces its accumulation in cells. Figure A1 (c) shows the mRNA content of HSP70 in mode-K cells. mRNA content of HSP70 in cells from the 39 °C treatment group was elevated compared with the 37 °C control group, but the difference was statistically non-significant ($p > 0.05$). The mRNA content of HSP70 in cells from the 41 °C and 43 °C treatment groups was elevated and the difference was significant ($p < 0.05$). mRNA levels of HSP70 in

cells from the 39 °C, 41 °C, and 43 °C treatment groups exhibited significant differences between each other, while the 37 °C and 39 °C treatment groups were non-significant, indicating that the three heat stress temperatures could represent different levels of heat stress. At the same treatment temperature, the mRNA content of HSP70 showed an increasing trend with increasing heat stress time, and the higher the heat stress temperature, the greater the increase in content. The mRNA content of HSP70 was not significantly different among the four heat stress treatments in the 39 °C treatment group. The mRNA content of HSP70 in the 41 °C and 43 °C treatment groups was significantly different after 6 h of heat treatment. Confirming that the mode-K cells underwent heat stress. This result also showed that different levels of heat stress and different heat treatment times led to changes in the mRNA content of HSP70 in mode-K cells; the greater the degree of heat stress and the longer the heat stress time, the more significant the effect.

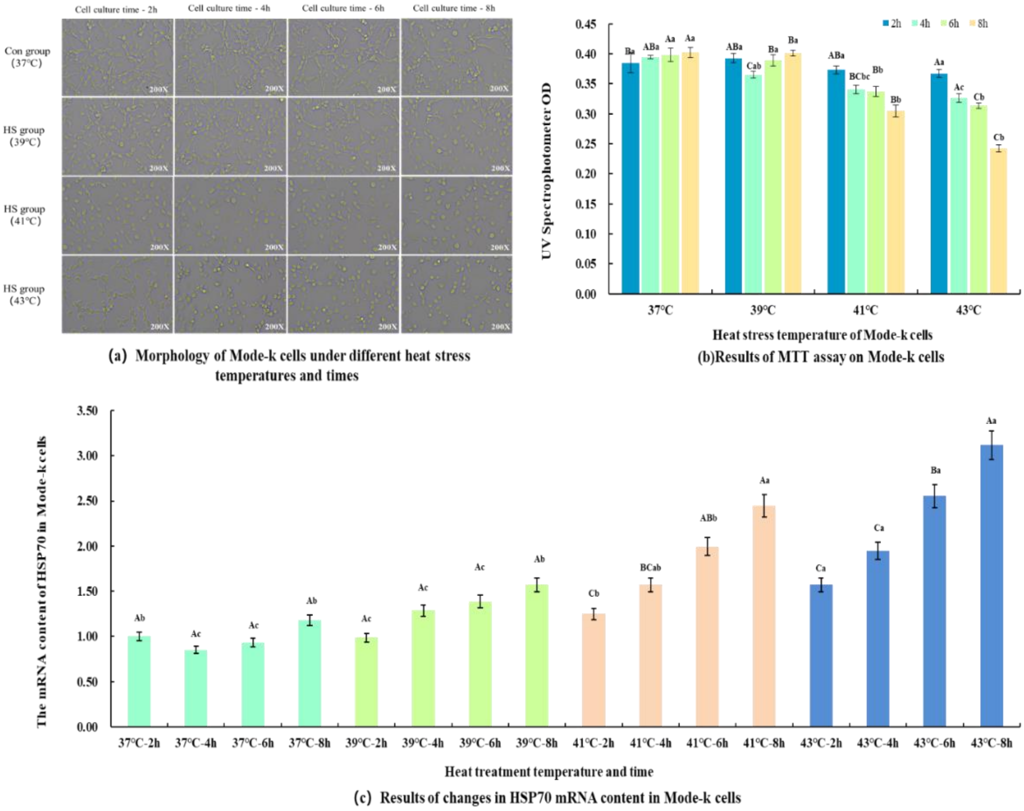

Figure. A1 Changes in morphology, cell viability and HSP70 mRNA content of Mode-k cells under different heat stress temperatures and times

Heat stress disturbs the body's metabolic system, leading to tissue damage and changes in enzyme activity and levels. Superoxide dismutase (SOD), which scavenges superoxide anion radicals, plays a crucial role in the oxidative and antioxidant homeostasis of organisms and is a central indicator of their antioxidant systems. Glutathione peroxidase (GSH-PX) can play an antioxidant role by specifically

catalyzing the reduction of hydrogen, thereby protecting the structure and intact function of cell membranes. It is also an important indicator for evaluating the antioxidant system of an organism. From Figure A2(a) and (b), it can be seen that the SOD and GSH-Px contents in the cells gradually decreased with the extension of treatment time under the same heat stress temperature. This difference was significant. As the heat-stress temperature increased, both antioxidant enzymes also showed significant differences between the different heat-stress temperatures. This indicates that mild, moderate, and severe heat stress can lead to oxidative stress in mode-k cells, resulting in a significant decrease in antioxidant enzyme activity. The decrease in GSH-PX content may also indicate that the integrity of the cell membrane is affected by heat stress. LDH is an important enzyme found in the cytoplasm that is released extracellularly when the cell membrane is damaged. As shown in Figure A2(c), the LDH content gradually increased with increasing treatment time at the same temperature. At the same time, as the degree of heat stress increased, its content also tended to increase, and all differences between the two were significant ($p < 0.05$). MDA is an oxidative end-product of lipid peroxidation caused by the action of free radicals in the body. Its high or low level reflects the degree of damage to the organism by free radical attack. The total antioxidant capacity (T-AOC) reflects the total level of various antioxidant macromolecules, small molecules and enzymes in the system. As shown in Figure A2(d) and (e), the MDA content showed a gradual increase with the increase in both heat stress temperature and heat treatment time, and the difference between different heat stress temperatures was significant. This indicated that heat stress caused lipid peroxidation and cell damage. At the same heat stress temperature, the difference in MDA content between 2 h and 4 h and between 6 h and 8 h was not significant, and the T-AOC of mode-K cells decreased with increasing heat stress temperature and heat treatment time. This indicated that heat stress causes oxidative stress in the cells.

Combining the five antioxidant evaluation indices, it was found that heat stress can cause a decrease in the antioxidant content and an increase in the pro-oxidant content in cells, in line with the results of previous studies [1-3]. Compared to the control group (37 °C), the change in the indicators became more significant as the degree of heat stress increased. Similarly, the more significant the difference between the indicators at the same temperature and at each heat-stress time, the more significant the difference. The significance of the difference in the indices initiated after 6 h of heat treatment at each temperature. *In summary*, the MTT cell viability assay, change in HSP70 content, and results of the five cellular antioxidant indicators were combined. The modelling time of heat stress for mode-k cells at 39 °C, 41 °C and 43 °C was determined as 6 h.

To confirm the successful modelling of different heat-stress temperature cell models under heat-stress conditions for 6 h, the study was carried out using real-time quantitative PCR on the relative expression of HSP27, and HSP90 genes. Relative expression of Claudin-1 and ZO-1 mRNAs was related to intercellular junctions. The relative expression of Bcl-2 and Bax genes was associated with apoptosis. Relative mRNA expression of TNF-α and IL-1β genes was related to inflammation, and

changes in the mitochondrial membrane potential were measured. The results are shown in Figure A2(f-g). HSP27, HSP70 and HSP90 are the three major classes of heat shock proteins. During the onset of and recovery from heat stress, it was primarily HSP70 that played a major role. HSP70 and HSP90 play a central role in the regulation of protein homeostasis, as well as in cell protection and repair and are general markers of cell injury. HSP27 is intrinsically anti-apoptotic. The mRNA content of HSP27 and HSP90 in mode-k cells increased with increasing heat stress temperature and showed significant differences between each other, indicating that heat stress did occur in the cells under 6 h of heat treatment, and to different degrees. Among the tight small-loop protein ZO-1, the closed junction-associated proteins were used as indicators to observe the function of the tight junction barrier and the permeability of various tissues. The claudin family of proteins is the most important of the many tight junction-associated transmembrane proteins and plays an important role in maintaining cell polarity and tight junction barrier functions. As shown in Figure 3(i) and (l), the mRNA content of both tight junction proteins decreased with increasing heat stress temperature, and the difference between them was significant, indicating that the tight junction proteins were "loosened" in mode-k cells under heat stress. Heat stress can weaken the immune status of the body [4-5] and cause oxidative stress. Oxidative stress can activate a variety of transcription factors, leading to the differential expression of a number of genes involved in inflammatory pathways. As shown in Figure A2(j) and (m), interleukin (IL-1β) and tumor necrosis factor (TNF-α) mRNA levels increased with increasing heat-stress temperature, indicating that heat-stress treatment leads to intracellular inflammation. Heat stress can also induce apoptosis. From Figure A2(h) and (k), we can see that the cell survival factor BCL-2 tends to decrease and the death regulator Bax tends to increase with an increase in heat stress, indicating that different degrees of heat stress can lead to apoptosis, and that apoptosis varies between different temperatures. The mitochondrial membrane potential is a common indicator of cellular health. When cells are normal, JC-1 enters the mitochondria through the mitochondrial membrane sex and forms polymorphs that emit red fluorescence at increased concentrations. In contrast, the mitochondrial membrane potential of apoptotic cells is de-J-ed, JC-1 is released from the mitochondria, and its concentration is reduced. As shown in Figure A2(n), with the increase in heat stress temperature, the offset of the cell population gradually increased and the amount of apoptosis gradually increased, further confirming that mode-k cells underwent a heat stress response under the 6 h heat stress treatment. The degree of heat stress gradually increased with increasing temperature. The combination of multiple indicators confirms that model of heat stress in mode-k cells at different temperatures was successfully constructed.

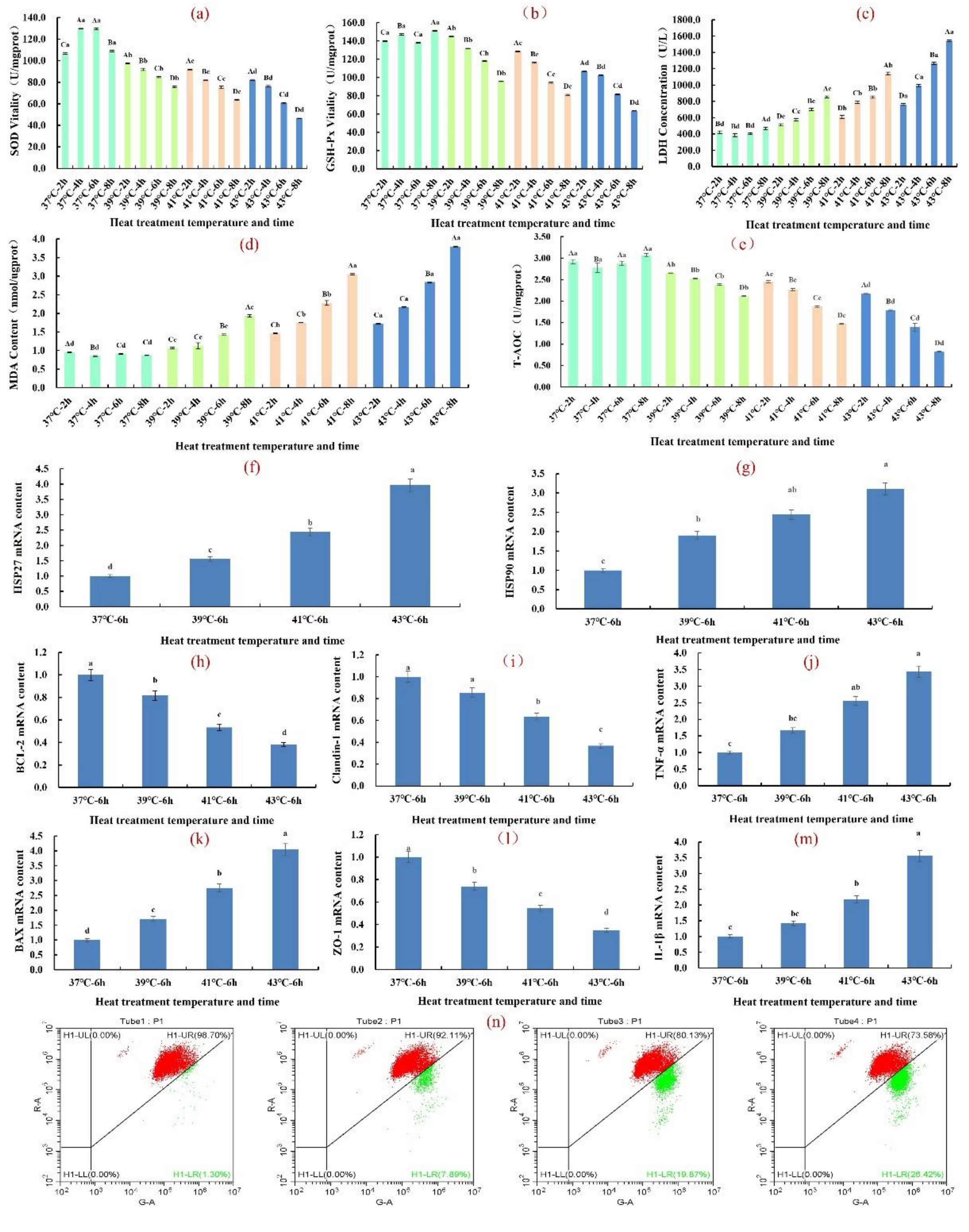

Figure. A2 Antioxidant indicators, heat shock protein, tight junction protein, inflammatory factor, apoptotic factor mRNA content and membrane potential ratio changes in Mode-k cells

3.2 Caco-2 cell model construction and heat stress fraction screening

Whether the fractions derived from mung beans have the same preventive and regulatory effects on heat stress in human-derived intestinal cells requires further confirmation. Therefore, Caco-2 cells were used in this study to corroborate these results. As shown in Figure A3(a), Caco-2 cells cultured at 37 °C showed irregular polygonal shapes, with the cells gradually contracting and the cell gap becoming

larger as the temperature increased. Cell viability decreased with increasing heat stress time at both 41 ℃ and 43 ℃. The changes in cell viability were more significant at different heat stress temperatures and heat treatment times after 6 h, Figure A3(b). This trend was similar to that observed for the mode-k cells. Figure A3(c) shows the results of mRNA content of HSP70 in Caco-2 cells. From the latter figure, it can be seen that the mRNA content of HSP70 gradually increased with an increase in heat stress temperature, while the higher the temperature, the greater the increase in mRNA content, the difference of which was significant. The mRNA content of HSP70 gradually increased with increasing heat stress time at the same temperature, and the change in content at the heat stress time of 6 h was significantly different ($p < 0.05$). The results showed that different levels of heat stress and different heat treatment times lead to an increase in the mRNA content of HSP70 in Caco-2 cells, indicating that the cells undergo a heat stress response, while the higher the heat stress temperature and the longer the time, the more significant the effect. The other modeling validation metrics are the same as Mode-K and the results trend the same as Mode-k.

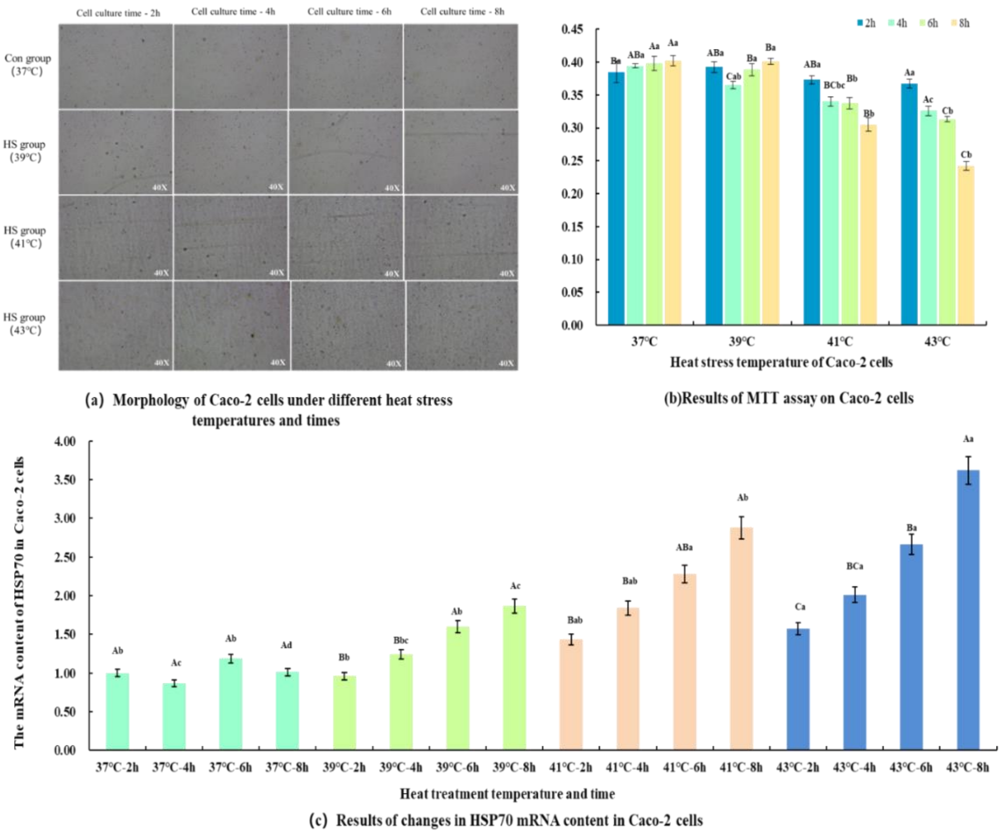

Figure. A3 Changes in morphology, cell viability and HSP70 mRNA content of Caco-2 cells under different heat stress temperatures and times

Figure A4(a-e) show the results of antioxidant indices in Caco-2 cells where it can be seen that under the same heat stress temperature, the SOD and GSH-Px content

in the cells showed a gradual decrease with the prolongation of heat stress time, while the difference in the content under different heat stress times was highly significant. The levels of both antioxidant enzymes decreased as the heat-stress temperature increased, and the differences were significant at different heat-stress temperatures. The levels of both antioxidant enzymes decreased as the heat-stress temperature increased, and the differences were significant at different heat-stress temperatures. LDH levels increased with increasing temperature and duration of heat stress, and were significantly different from each other ($p < 0.05$). With an increase in both heat stress temperature and heat stress time, the MDA content showed a gradual increase. There was little difference between 39 °C and 37 °C, with a significant difference in MDA content at 6 h of heat stress in the 39 °C treatment group. The differences in MDA content between each other was significant at both 41 °C and 43 °C. The T-AOC of Caco-2 cells decreased with increasing heat-stress temperature and heat-treatment time. Significant differences in MDA content emerged in the 39 °C treatment group after 6 h of heat stress. The differences in changes in T-AOC between each other at both 41 °C and 43 °C were significant.

After combining the five antioxidant evaluation indices, it was found that heat stress can cause a decrease in antioxidant content and an increase in pro-oxidant content in Caco-2 cells. Heat treatment causes heat stress and oxidative stress in Caco-2 cells. As the heat stress temperature increased, the more significant the changes in the indicators were compared to the control group (37 °C). The longer the duration of heat stress at the same temperature, the more significant the difference in the indicators was. The changes in the five indicators due to different heat-stress temperatures and heat-stress times were combined. All five antioxidant indicators were significantly different in the 39 °C treatment group at 6 h of heat stress and in the 41 °C and 43 °C treatment groups at 4 h of treatment. In summary, it can be seen that the individual indexes of Caco-2 cells changed in the same way as Mode-k. Compared with the 37 °C control group, most of the indicators at different heat stress temperatures at 2 h and 4 h of heat stress showed significant changes. However, in combination with the changes in cell viability, HSP70 mRNA content, and antioxidant indicators, almost all indicators changed significantly after 6 h of heat stress, making the effect of different levels of heat stress more obvious. Since the difference between the different levels of heat stress was significant, the modelling time of heat stress for Caco-2 cells was also determined to be 6 h.

Figure A4. Antioxidant indicators, heat shock protein, tight junction protein, inflammatory factor, apoptotic factor mRNA content and membrane potential ratio changes in Caco-2 cells Similarly, cellular model building for Caco-2 cells under heat stress for 6h was also validated. These results are shown in Figure A4(f-n). The mRNA content of HSP27 and HSP90 in Caco-2 cells increased with increasing heat-stress temperature. Compared to the 37 °C control, the mRNA levels of the three heat shock proteins were elevated, but not significantly different at 39 °C, and significantly different at both 41 °C and 43 °C. This indicates that heat stress occurred in cells before the 6 h mark of heat treatment and to different degrees. The mRNA content of both the tight junction proteins ZO-1 and claudin decreased with increasing heat stress

temperature, indicating that heat stress for 6 h loosened the tight junction proteins and significantly affected cell membrane integrity. The RNA content of the inflammatory factors IL-1β and TNF-α increased with increasing heat stress temperature, suggesting that heat stress treatment led to inflammation in Caco-2 cells. The cell survival factor BCL-2 showed a decreasing trend with increasing heat stress temperature, while the death regulator Bax showed an increasing trend, indicating that different levels of heat stress can lead to apoptosis, whereby the levels of apoptosis varied at different temperatures. It was also possible to find non-significant differences in the indicators at 39 °C compared to 37 °C and significant differences in the indicators at 41 °C and 43 °C, indicating that different levels of heat stress were met. In the mitochondrial membrane potential assay, the excursion of the cell population gradually increased with increasing heat-stress temperature, and the amount of apoptosis gradually increased. This further confirmed that Caco-2 cells underwent a heat stress response during the 6 h heat stress treatment. The degree of heat stress increased with increasing temperature. In conclusion, we successfully constructed a heat stress model for Caco-2 cells at different temperatures.

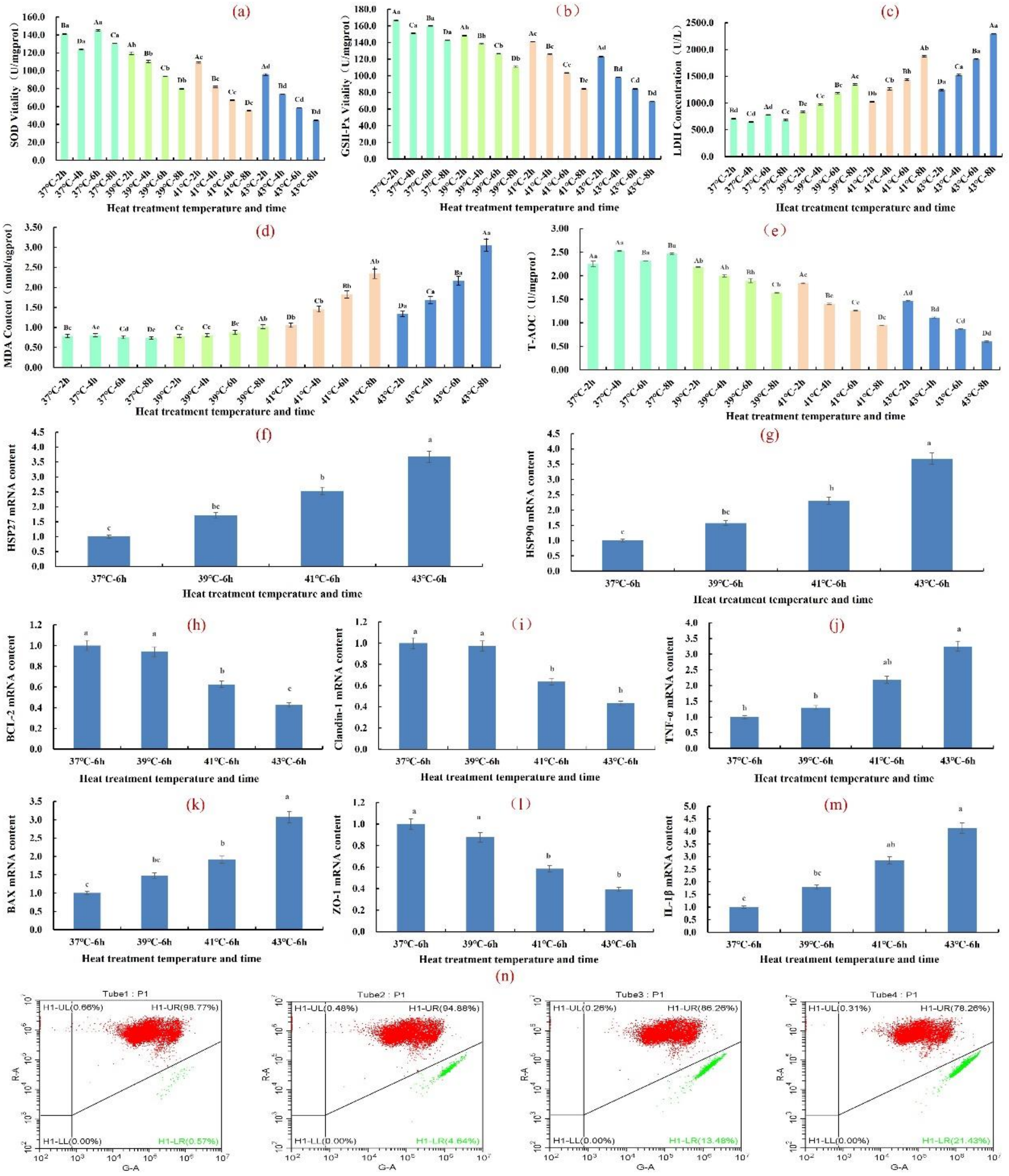

Figure. A4 Antioxidant indicators, heat shock protein, tight junction protein, inflammatory factor, apoptotic factor mRNA content and membrane potential ratio changes in Caco-2 cells

References：

1. Frijhoff J, Winyard PG, Zarkovic N, Davies SS, Stocker R, Cheng D, et al. Clinical relevance of biomarkers of oxidative stress. Antioxid Redox Signal. (2015) 23:1144–70. doi: 10.1089/ars.2015.6317

2. Das A. Heat stress-induced hepatotoxicity and its prevention by resveratrol in rats. Toxicol Mech Methods. (2011) 21:393–9. doi: 10.3109/15376516.2010.550016

3. Li X, Y ang Y , Liu S, Y ang J, Chen C, Sun Z. Grape seed extract supplementation

attenuates the heat stress-induced responses of jejunum epithelial cells in Simmental × qinchuan steers. Br J Nutr. (2014) 112:347–57. doi: 10.1017/s0007114514001032

4. Sordillo LM, Aitken SL. Impact of oxidative stress on the health and immune function of dairy cattle. Vet Immunol Immunopathol. (2009) 128:104–9. doi: 10.1016/j.vetimm.2008.10.305

5. V andana GD, Bagath M, Sejian V , Krishnan G, Beena V , Bhatta R. Summer season induced heat stress impact on the expression patterns of different toll-like receptor genes in Malabari goats. Biol Rhythm Res. (2018) 50:466–82. doi:10.1080/09291016.2018.1464638
